# Causal link between folic acid and recurrent aphthous ulcers: A two-sample Mendelian randomization study

**DOI:** 10.1097/MD.0000000000049125

**Published:** 2026-06-12

**Authors:** TieXin Gao, TianYang Chen, YuLong Xu, LiuXin He, YanJun Hou, MinYong Gong

**Affiliations:** a Department of Oncology, Hospital of Traditional Chinese Medicine of Jiujiang, Jiujiang, China; b Jiangxi University of Chinese Medicine, Nanchang, China; c Changchun University of Chinese Medicine, Changchun, China.

**Keywords:** causal association research, folic acid, Mendelian randomization, micronutrients, recurrent aphthous ulcers

## Abstract

Observational studies have suggested associations between micronutrient status and recurrent aphthous ulcers (RAU); however, the causal nature of these relationships remains unclear. This study aimed to evaluate the genetically predicted associations between circulating micronutrients and RAU using a Mendelian randomization (MR) approach. We conducted a two-sample MR analysis using genome-wide association study (GWAS) summary statistics from individuals of European ancestry. Genetic instruments for 15 micronutrients, including folic acid, zinc, and vitamin B12, were obtained from the IEU OpenGWAS database, while RAU outcome data (5394 cases) were derived from the FinnGen consortium. The inverse variance weighted (IVW) method was used as the primary analysis, complemented by weighted median, MR-Egger, and sensitivity analyses to assess heterogeneity and horizontal pleiotropy. The IVW analysis indicated a nominally significant association between genetically predicted folic acid levels and RAU risk (odds ratio [OR] = 2.05, 95% confidence interval [CI]: 1.20–3.50, *P* = .009). Other MR methods showed effect estimates in a consistent direction but with wide CIs crossing the null. No statistically significant associations were observed for the remaining 14 micronutrients. Sensitivity analyses did not provide evidence of substantial heterogeneity or horizontal pleiotropy. This MR study provides genetic evidence supporting a potential association between genetically predicted folic acid levels and the risk of RAU, primarily driven by the IVW analysis. Given the nominal significance and methodological limitations, these findings should be interpreted cautiously and do not imply direct clinical or dietary recommendations. Further studies are warranted to confirm these results and to elucidate the underlying biological mechanisms.

## 1. Introduction

Recurrent aphthous ulcers (RAU), a common condition of the oral mucosa, are primarily caused by an overreactive immune response involving numerous immunological factors. These factors, such as interleukins (IL-1β, IL-2, IL-4, IL-5, IL-6, IL-10, IL-12), interferon (IFN-γ), and tumor necrosis factor (TNF-α), intensify inflammation and cause local tissue damage.^[1–4]^ The prevalence of RAU varies between 1.4% and 21.4% according to retrospective demographic studies across different countries and regions, with a positive family history in 24 to 46% of cases, highlighting its significant public health burden. Previous studies indicate that RAU is more common among adolescents and young adults and tends to occur more frequently in females, which further informs the public health perspective on this condition.^[3,5–7]^ RAU manifests in 3 forms – mild, moderate, and herpetiform – with mild RAU being the most common (about 80% of cases). It typically presents as painful ulcers on non-keratinized mucosa that heal without scarring in 7 to 14 days but can disproportionately affect quality of life.^[8]^ For example, RAU can negatively impact eating, speaking, sleep, school or work attendance, and psychosocial well-being.

Observational evidence has linked RAU to micronutrient deficiencies.^[9,10]^ For example, zinc deficiency may contribute to RAU pathogenesis by promoting immune factors such as interleukin-1, interleukin-6, and tumor necrosis factor-α, resulting in cytokine dysregulation.^[11–13]^ Vitamin B12, which is essential for cellular functions, exhibits polymorphisms in transporters like transcobalamin II that are associated with gastrointestinal ulcers; its deficiency can also lead to complex phenotypic changes.^[14–17]^ Folic acid deficiency, primarily due to inadequate dietary intake, has been implicated in various health issues, including impaired deoxyribonucleic acid (DNA) synthesis and repair. These impairments may increase mucosal vulnerability and cancer risk, although excessive folic acid intake might mask vitamin B12 deficiency or alter DNA methylation.^[8,15,18–23]^ However, most prior studies focus on deficiency states – particularly low levels of folate, iron, or vitamin B12 – while evidence regarding elevated folic acid levels and RAU remains limited. Clarifying this gap justifies adopting a causal genetic approach.^[9,24,25]^

Despite these associations, causality remains uncertain due to limitations in observational studies, such as confounding by diet, lifestyle, systemic diseases, or reverse causation, which hinder clear inferences. Mendelian randomization (MR) is an appropriate method to address this, using genetic variants as instrumental variables in “natural experiments” to evaluate causal effects of exposures like micronutrients.^[26–28]^ By leveraging randomly allocated genetic variations independent of confounders, MR minimizes bias common in traditional epidemiology. This study uses two-sample MR to investigate causal associations between 15 micronutrients (copper, calcium, iron, magnesium, potassium, selenium, zinc, carotene, folic acid, vitamin A, vitamin B6, vitamin B12, vitamin C, vitamin D, and vitamin E) and RAU in European populations. We hypothesize that genetically predicted elevated folic acid levels may be associated with increased RAU risk.

## 2. Methods

### 2.1. Ethical considerations

This study utilized publicly available summary statistics from genome-wide association study (GWAS) consortia and the FinnGen cohort, with no individual-level data analyzed. Therefore, ethical approval was waived by the Institutional Review Board of Jiangxi University of Chinese Medicine, as it did not involve direct human subjects. No informed consent was required for the same reason. The study adhered to the principles of the Declaration of Helsinki (revised in 2013).

### 2.2. Study design

**Figure 1.**
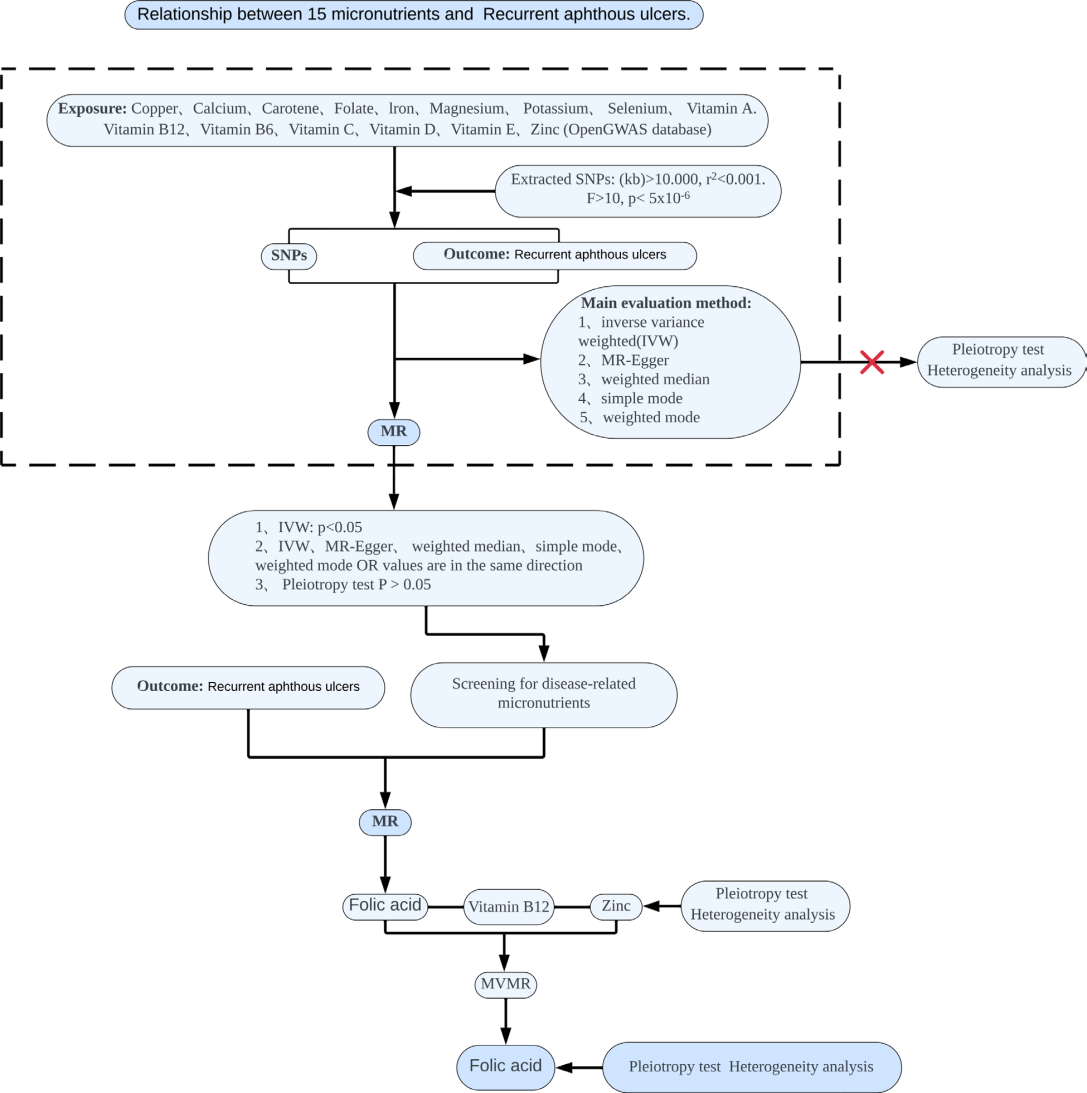
Summary of the MR study design for the relationship between 15 micronutrients and RAU. IVW = inverse variance weighting, MR = Mendelian randomization, MVMR = multivariate Mendelian randomization, RAU = recurrent aphthous ulcers, SNPs = single nucleotide polymorphisms.

**Figure 2.**
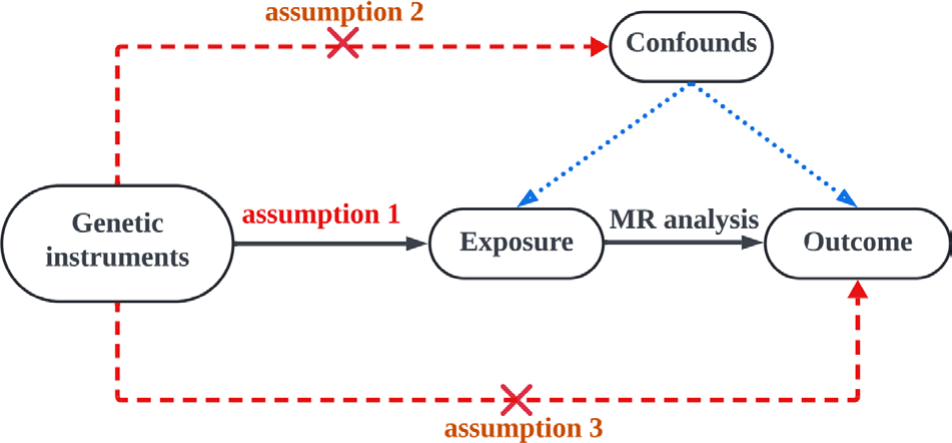
The first hypothesis is that the selected SNPs are significantly associated with the exposure factor (micronutrients); the second hypothesis is that the SNPs must be independent of potential confounders between the exposure and the outcome; and the third hypothesis is that the SNPs are not directly related to the RAU but only causally related through the micronutrients. RAU = recurrent aphthous ulcers, SNPs = single nucleotide polymorphisms.

Figure 1 depicts the study design. Three fundamental assumptions underlie this MR analysis, as shown in Figure 2: the selected single nucleotide polymorphisms (SNPs) are strongly associated with the exposure factor (micronutrients); the SNPs are independent of potential confounders between exposure and outcome; and the SNPs have no direct effect on RAU but influence it only through the micronutrients.

### 2.3. Data source

**Figure 3.**
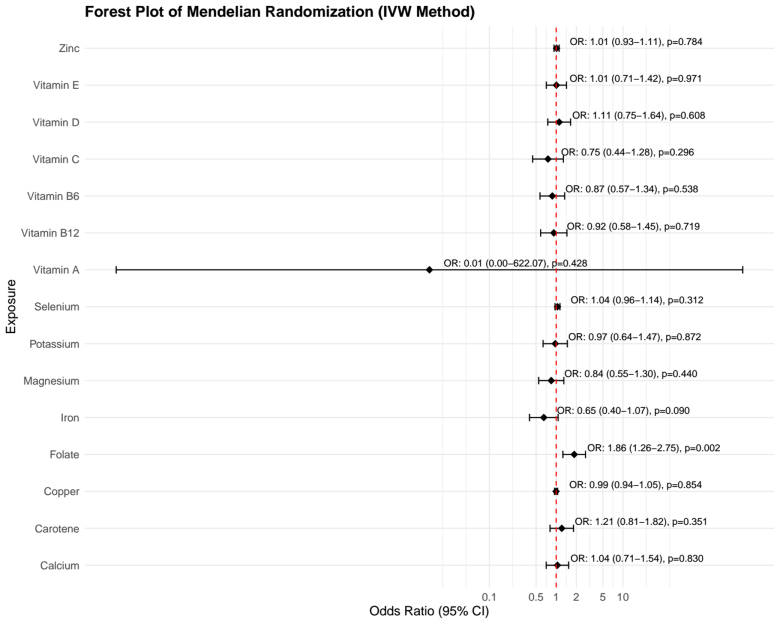
MR analysis results of exposures (copper, calcium, iron, magnesium, potassium, selenium, zinc, carotene, folate, vitamins A, B6, B12, C, D, and E) and outcome (RAU). Five methods: inverse variance weighting (IVW), weighted median, MR-Egger, simple mode, and weighted mode. MR = Mendelian randomization, RAU = recurrent aphthous ulcers.

We selected 15 micronutrients (copper, calcium, iron, magnesium, potassium, selenium, zinc, carotene, folic acid, vitamin A, vitamin B6, vitamin B12, vitamin C, vitamin D, and vitamin E) based on their reported associations with RAU or oral health in observational literature. GWAS summary statistics for these micronutrients were obtained from the IEU OpenGWAS database. RAU GWAS data (n = 5394 cases) were sourced from the FinnGen Biobank, which provides detailed information on participants, genetic analysis, imputation, and quality control. All datasets are from European ancestry populations. The exposure (micronutrient GWAS) and outcome (RAU GWAS) datasets represent independent samples, as the micronutrient data are primarily from UK Biobank or other European cohorts, while FinnGen is based on Finnish participants with no sample overlap. Table 1 provides specific data details. No institutional review board approval or informed consent was required, as all data were publicly available. The analysis of the MR findings is shown in Figure 3.

**Table 1:** Exposure data from the OpenGWAS database: copper, calcium, iron, magnesium, potassium, selenium, zinc, carotene, folate, vitamins A, B6, B12, C, D, and E.

	Trace element	GAWS ID	Sample size
Exposure	Copper	ieu-a-1073	2603
Exposure	Calcium	ukb-b-8951	64,979
Exposure	Carotene	ukb-b-16202	64,979
Exposure	Folate	ukb-b-11349	64,979
Exposure	Iron	ukb-b-20447	64,979
Exposure	Magnesium	ukb-b-7372	64,979
Exposure	Potassium	ukb-b-17881	64,979
Exposure	Selenium	ieu-a-1077	2603
Exposure	Vitamin A	ukb-b-9596	460,351
Exposure	Vitamin B12	ukb-b-19524	64,979
Exposure	Vitamin B6	ukb-b-7864	64,979
Exposure	Vitamin C	ukb-b-19390	64,979
Exposure	Vitamin D	ukb-b-18593	64,979
Exposure	Vitamin E	ukb-b-6888	64,979
Exposure	Zinc	ieu-a-1079	2603
Outcome	Recurrent aphthous ulcers	finngen_R12_K11_APHTA_RECUR_INCLAVO	5394

Outcome data from the FinnGen database: recurrent aphthous ulcers.

### 2.4. Instrumental variable processing

SNPs associated with each micronutrient were selected as instrumental variables based on the following criteria to ensure robustness and reproducibility: a genome-wide significance threshold of *P* < 5 × 10^−6^ (relaxed from the conventional *P* < 5 × 10^−8^ to include more instruments for micronutrients with limited genome-wide significant SNPs), as commonly used in MR studies of circulating nutrients^[29,30]^; linkage disequilibrium clumping (*r*^2^ < 0.001, window = 10,000 kb) to ensure independence of SNPs; exclusion of SNPs associated with the outcome (RAU) or known confounders (e.g., body mass index, smoking status) as identified via the PhenoScanner database; calculation of *F*-statistics for each SNP, with those having *F* < 10 excluded to mitigate weak instrument bias.

### 2.5. Statistical analysis

All analyses were performed using R software (version 4.4.1) and the TwoSampleMR package.^[31]^ To ensure accurate estimation, exposure and outcome data were harmonized by aligning effect alleles. Palindromic SNPs with intermediate allele frequencies (0.42 < EAF < 0.58) were excluded to prevent strand ambiguity. We employed multiple MR methods: inverse variance weighted (IVW) as the primary method (prioritized because it provides the most efficient estimates assuming all instruments are valid).^[32]^ supplemented by weighted median, MR-Egger, simple mode, and weighted mode. Causal estimates represent the change in log-odds of RAU per standard deviation increase in the circulating micronutrient level. Sensitivity analyses included Cochran *Q* test for heterogeneity, MR-Egger intercept test and MR pleiotropy residual sum and outlier global test for horizontal pleiotropy,^[33,34]^ and leave-one-out analysis. Results from the IVW method were considered the primary finding, with consistency across other methods and sensitivity analyses supporting robustness. Multivariable MR (MVMR) was used to assess the independent effect of folic acid, adjusting for zinc and vitamin B12. For the MVMR, we extracted SNPs associated with any of the 3 exposures (folic acid, zinc, vitamin B12) and assessed collinearity between the genetic instruments, which was found to be low.^[35,36]^ The 15 micronutrients were pre-specified based on prior observational literature linking them to RAU or oral health.

## 3. Results

### 3.1. MR analysis

**Figure 4.**
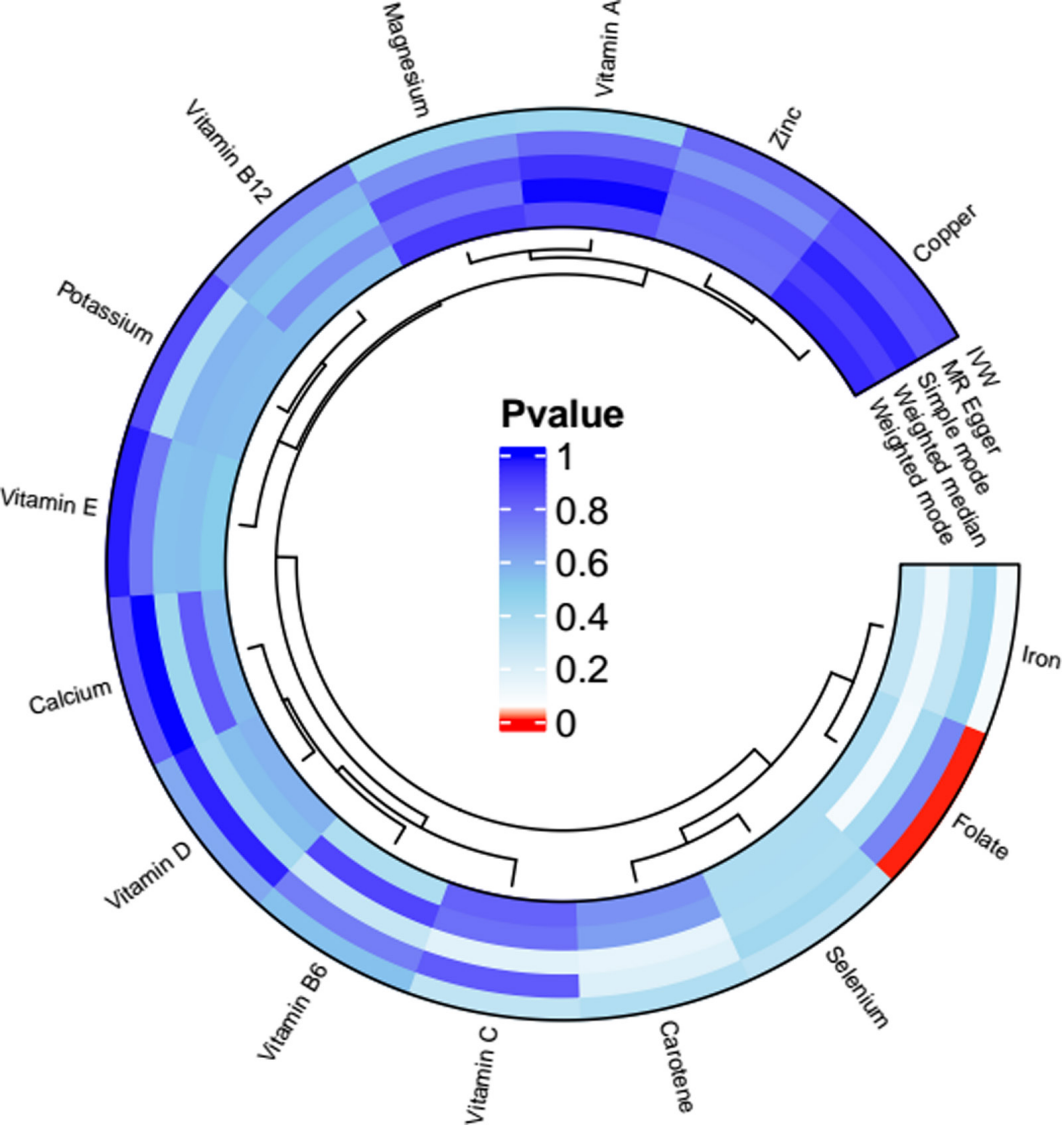
Forest plot of Mendelian randomization analyses for 15 micronutrients on recurrent aphthous ulcers (RAU). The odds ratios (ORs) and 95% confidence intervals (CIs) were derived from the inverse variance weighted (IVW) method. A causal effect is considered statistically significant if the 95% CI does not cross the vertical line (OR = 1). Only folic acid showed a nominally significant causal association (*P* < .05) that did not survive multiple testing correction.

We performed a two-sample MR analysis to assess the causal effects of 15 micronutrients on RAU. The primary IVW method suggested a potential positive causal effect of folic acid on RAU risk, with an odds ratio (OR) of 1.861 (95% confidence interval [CI]: 1.261–2.748, *P* = .002) per standard deviation increase in folic acid levels. **Statistical significance was observed only with the IVW method, while other MR methods (weighted median, MR-Egger, simple mode, weighted mode) showed a consistent direction but wide CIs including the null value. No significant causal associations were observed for the other 14 micronutrients after multiple testing correction. Figure 4 presents a consolidated forest plot of the IVW results for all micronutrients. Sensitivity analyses for folic acid indicated no evidence of horizontal pleiotropy (MR-Egger intercept *P* = .711; MR pleiotropy residual sum and outlier global test *P* = .266) or heterogeneity (Cochran *Q P* > .05). The F-statistics for all SNPs used as instruments for folic acid exceeded 10, indicating a low risk of weak instrument bias (Supplementary Files 1–7, Supplemental Digital Content 1–7).

**Figure 5.**
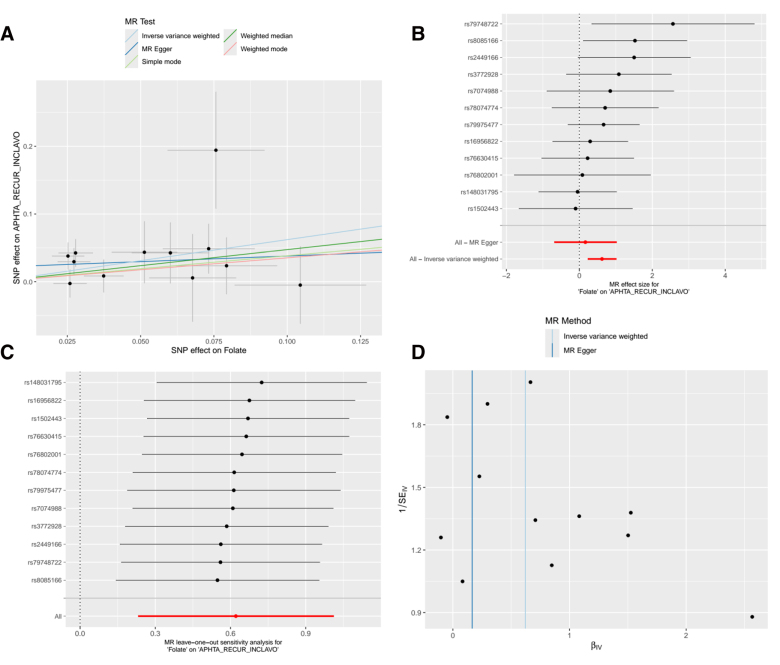
Mendelian randomization analysis of the causal relationship between folate and RAU. (A) Scatterplot of the causal relationship between folate and RAU. The slope of the straight line indicates the magnitude of the causal relationship. Black dots represent the genetic tools included in the main Mendelian randomization analysis. (B) Forest plot showing the causal effect of each SNP on RAU risk. (C) Leave-one-out plot of the causal relationship between folate and RAU risk. (D) Funnel plot showing SNP heterogeneity. RAU = recurrent aphthous ulcers, SNP = single nucleotide polymorphism.

The R software was used to determine micronutrients linked to disease. Folic acid was linked to RAU and showed a pleiotropy *P*-value of .266 (*P* > .05), according to the analysis (Supplementary File S2, Supplemental Digital Content 2). Folic acid, a vitamin associated with RAU, was subjected to MR analysis (Supplementary File S3, Supplemental Digital Content 3). Folic acid was found to be substantially linked with RAU by the random effects inverse variance weighting (IVW) analysis (*P* = .002, OR = 1.861, 95% CI = 1.261–2.748). Testing for pleiotropy (Supplementary File S4, Supplemental Digital Content 4) and heterogeneity (Supplementary File S5, Supplemental Digital Content 5) revealed no significant pleiotropy or heterogeneity, with p-values over 0.05. According to the results of outlier detection, no outliers were found in the individual SNP outlier detection (Supplementary File S7, Supplemental Digital Content 7), and the overall *P*-value for all outlier tests was > .05 (Supplementary File S6, Supplemental Digital Content 6). Furthermore, SNP results pertaining to exposure and outcome factors were consistent across the 5 methodologies employed in the analysis, as shown by scatter plots. The MR results were not significantly impacted by the removal of any one SNP, according to the leave-one-out sensitivity analysis. A symmetrical distribution was shown by the funnel plot, indicating no discernible bias (Fig. 5).

### 3.2. Multivariate MR analysis

**Figure 6.**
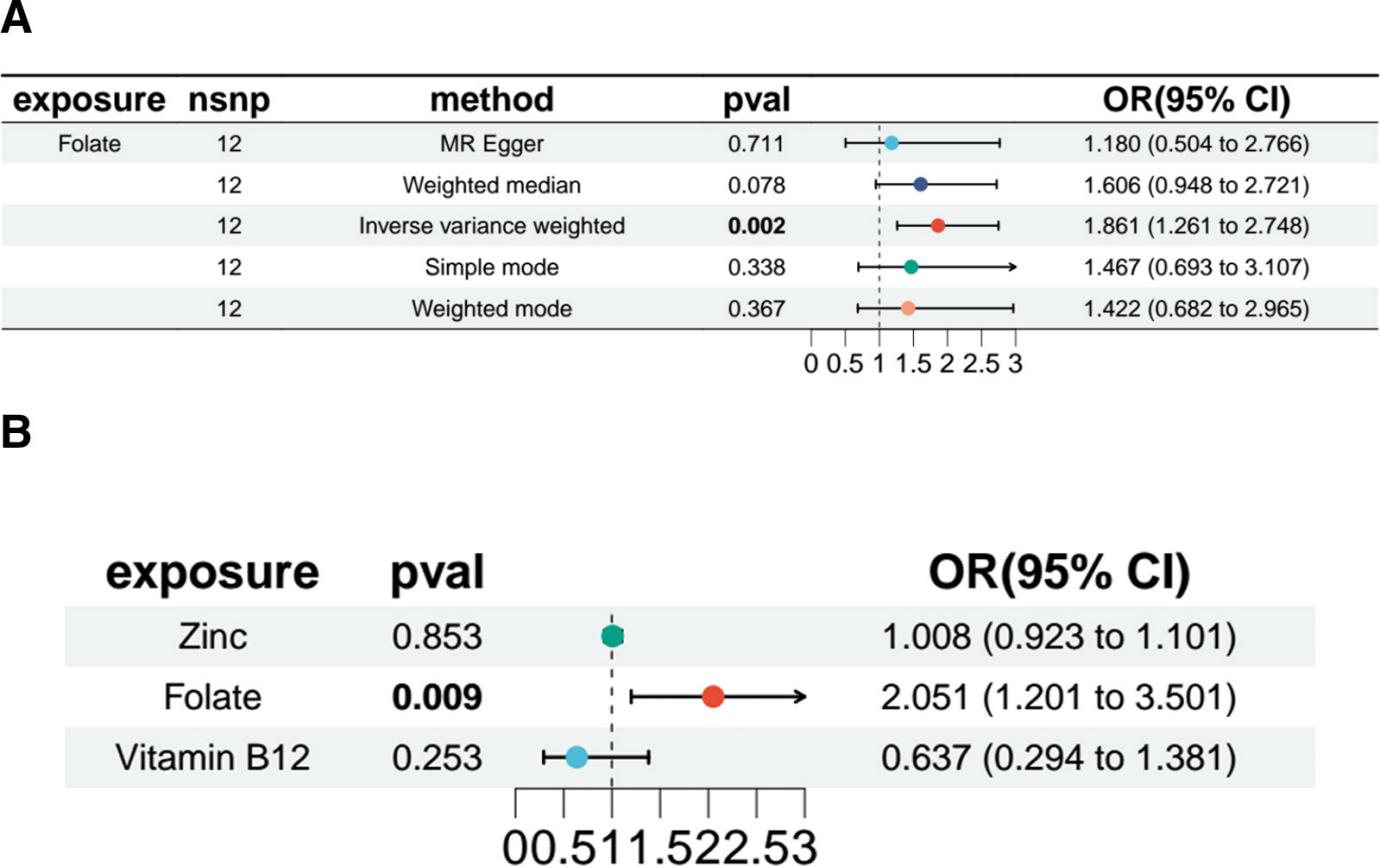
Forest plot. (A) Forest plot of Mendelian randomization analysis of folic acid with inverse variance weighting (IVW), weighted median, MR-Egger, simple mode, and weighted mode. (B) Forest plots for inverse variance-weighted Mendelian randomization analysis of zinc, vitamin B12, and folic acid. MR = Mendelian randomization.

In a multivariable MR model adjusting for zinc and vitamin B12 – two micronutrients frequently implicated in RAU pathogenesis – the point estimate for folic acid remained elevated (OR = 2.01, 95% CI: 1.201–3.501, *P* = .009), suggesting a potential independent effect. Neither zinc (OR = 1.008, *P* = .853) nor vitamin B12 (OR = 0.637, *P* = .253) showed a significant association in this model (Supplementary File S8, Supplemental Digital Content 8). Tests for heterogeneity and pleiotropy were non-significant (all *P* > .05) (Fig. 6) (Supplementary File S9, Supplemental Digital Content 9).^[37]^

## 4. Discussion

This MR study investigated the causal relationships between 15 micronutrients and RAU. Our primary finding was a nominally significant association between genetically predicted higher folic acid levels and an increased RAU risk using the IVW method. This suggests a potential genetically predicted association rather than definitive causal or clinical effects, given that only the IVW analysis reached statistical significance. This finding challenges the conventional view of folic acid as a purely protective nutrient in this context and warrants careful interpretation.^[38]^

The discrepancy where significance was reached only with the IVW method, but not with other supplementary methods whose estimates were imprecise (wide CIs), is not uncommon in MR studies. The IVW method provides the greatest statistical power when the instrumental variables are valid. The consistent directional effect across all methods and the absence of detectable pleiotropy in sensitivity analyses lend some support to the robustness of the primary IVW result. However, the lack of statistical significance in other methods necessitates caution.

When compared with existing literature, our finding for folic acid appears novel. Previous observational studies have often focused on folate deficiency rather than elevated levels, whereas our genetic approach suggests a potential risk from higher genetically predicted levels.^[9,24,25]^The null findings for other micronutrients, such as zinc and vitamin B12, which have been previously implicated in observational studies, highlight the possibility that those associations may have been influenced by confounding or reverse causation. Our MR analysis does not support a causal role for these nutrients in RAU development.

The formation of DNA depends on folic acid, a water-soluble vitamin.^[39]^ It is an essential component of amino acid metabolism and nucleic acid synthesis, acting as a coenzyme or cosubstrate in one-carbon transfer. According to an intervention trial, uracil misincorporation and promoter methylation in rectal mucosal DNA were impacted by high-dose folic acid in patients with a history of colorectal adenomas.^[39–42]^ These mechanisms, drawn from studies on cancer and pregnancy, are not RAU-specific and involve exposure scenarios differing from genetically predicted folate levels; thus, they are speculative in the context of RAU.

Dietary consumption accounts for the bulk of folic acid in the human body. Its ability to prevent neural tube abnormalities, anemia, cardiovascular disorders, cognitive decline, and several types of cancer is well known.^[43–45]^ Higher folic acid levels aren’t always advantageous, though.^[46]^ Excessive folic acid is divided into 3 categories by the NIH workshop: unmetabolized folic acid (UMFA), high folic acid status, and excessive consumption.^[47]^ According to a randomized double-blind study, after 5 years, people taking 1 mg/day of folic acid supplements had more advanced lesions and numerous adenomas, along with a greater incidence of prostate cancer. This implies that patients with adenomas may be more susceptible to numerous or advanced polyps if they use high folic acid supplements.^[48]^ Dihydrofolate (DHF) buildup can result from high folic acid intake. Enzymatic activity in intestinal mucosal cells converts folic acid to 5-methyl tetrahydrofolate as part of the regular absorption mechanism. Excessive folic acid, on the other hand, avoids this typical absorption process, is taken up straight as folic acid, and is transformed into DHF in tissues. Thymidylate synthase and methylene tetrahydrofolate reductase activities are inhibited by the rise in DHF during one-carbon metabolism.^[24,48,49]^ According to an intervention trial, uracil misincorporation and promoter methylation in rectal mucosal DNA were impacted by high-dose folic acid in patients with a history of colorectal adenomas.^[50]^ Overdosing on folic acid can cause gastrointestinal problems and immune system dysfunction, which can result in oral and stomach ulcers.^[51,52]^ Consuming too much folic acid raises the risk of developing cancer.^[53,54]^ A high consumption of synthetic folic acid raises DNA methylation levels in white blood cells and colon tissue and encourages carcinogenesis in the colonic mucosa. High folic acid consumption raises the risk of rectal mucosa carcinogenesis in Kirsten rat tumor models, according to another review article.^[55–58]^ High folic acid levels have been linked to an increased risk of gestational diabetes mellitus, according to a Chinese cohort study. An increased risk of gestational diabetes is linked to longer supplementation duration, higher folic acid consumption, and long-term use. Furthermore, the risk of gestational diabetes is greatly raised when elevated folic acid levels are paired with an imbalance in vitamin B12 levels.^[46,59–62]^

Vitamin B12 has been implicated in the pathophysiology of RAU.^[25,63]^ Transcobalamin is required for cellular uptake of vitamin B12,^[64]^ and excessive folic acid intake may reduce holo-transcobalamin availability, thereby impairing vitamin B12 bioavailability and increasing homocysteine and methylmalonic acid levels.^[65]^ Experimental evidence further suggests that high folic acid exposure under conditions of vitamin B12 deficiency may promote oxidative stress and inflammatory responses.^[66]^ Therefore, one possible explanation is that excessive folic acid may indirectly contribute to RAU by disturbing vitamin B12 metabolism, particularly in individuals with underlying vitamin B12 insufficiency. However, because our MR analysis did not detect a significant association between genetically predicted vitamin B12 levels and RAU risk, this mechanism should be considered speculative and requires further investigation.

Our study has several limitations that must be acknowledged. First, the significance of the folic acid-RAU association did not withstand strict correction for multiple testing, increasing the possibility of a false-positive finding. Second, while sensitivity analyses showed no evidence of pleiotropy, we cannot definitively rule out this potential bias. Third, our analysis was restricted to individuals of European ancestry, limiting the generalizability of our findings to other populations. Fourth, we were unable to explore non-linear relationships between micronutrient levels and RAU risk. Fifth, the distinction between genetically predicted micronutrient levels and actual dietary intake or supplementation must be noted. Finally, the GWAS data for some micronutrients had relatively small sample sizes, which may have limited our power to detect genuine causal effects for other nutrients.

## 5. Conclusion

In conclusion, this two-sample MR analysis suggests a potential association between genetically predicted higher folic acid levels and an increased risk of RAU, with statistical significance observed only in the IVW analysis. No convincing genetic evidence was found to support causal roles for the other 14 micronutrients examined, including zinc and vitamin B12. Importantly, genetically predicted micronutrient levels reflect lifelong genetic predisposition and are not equivalent to dietary intake or supplementation. Therefore, these findings should not be interpreted as evidence to guide clinical management or folic acid supplementation practices. Instead, they provide a genetic epidemiological perspective that highlights the complexity of folate-related biology in RAU and underscores the need for further mechanistic and population-based studies in diverse cohorts.

## Acknowledgments

Confirm all named individuals have given permission.

## Author contributions

**Conceptualization:** MinYong Gong.

**Data curation:** TieXin Gao, TianYang Chen.

**Investigation:** TieXin Gao, TianYang Chen.

**Methodology:** MinYong Gong.

**Software:** TieXin Gao, TianYang Chen.

**Visualization:** YuLong Xu, LiuXin He, YanJun Hou.

**Writing – original draft:** TieXin Gao, TianYang Chen, YuLong Xu, LiuXin He, YanJun Hou.

**Writing – review & editing:** TieXin Gao, TianYang Chen, MinYong Gong.


















